# Resilience and ART Adherence Among a Sample of Racially and Ethnically Diverse Sexual Minority Men With HIV

**DOI:** 10.1155/arat/8199608

**Published:** 2025-08-12

**Authors:** Stephanie A. Meyers-Pantele, Jonathan L. Helm, Michael Miller-Perusse, Junye Ma, Keith J. Horvath

**Affiliations:** ^1^Division of Infectious Diseases and Global Public Health, Department of Medicine, University of California San Diego, La Jolla, California, USA; ^2^Department of Psychology, San Diego State University, San Diego, California, USA; ^3^Joint Doctoral Program in Clinical Psychology, Department of Psychology, San Diego State University/ University of California San Diego, San Diego, California, USA

## Abstract

While racial disparities in HIV antiretroviral treatment (ART) adherence and viral suppression among sexual minority men (SMM) with HIV persist, resilience may serve as an important protective factor. There is, however, a dearth of research exploring the longitudinal associations between resilience and ART adherence among this group. As such, the current study examined prospective associations, including the between- and within-person effects, between resilience and ART adherence among racially diverse SMM with HIV. Data were drawn from *Thrive With Me* (TWM), a randomized controlled trial of an mHealth intervention targeting ART adherence among SMM. Generalized estimating equations (GEEs) models examined longitudinal associations, including between- and within-person effects, between resilience scores and self-reported 30-day ART adherence, dichotomized as optimal (≥ 90% of doses) versus suboptimal (< 90% of doses) across the 17-month study timeframe, while controlling for covariates. Among 401 SMM with HIV that completed the TWM baseline assessment (*M*_age_ = 39.1 years, Standard Deviation = 10.8), 59.9% self-identified as Black/African American. In GEE models, resilience scores were prospectively associated with optimal 30-day ART adherence (*b* = 0.06, *β* = 0.38, *p* < 0.001), at the between-person level, above the effects of covariates. In moderation analyses, resilience scores were associated with optimal ART adherence among Black/African American SMM but not among those identifying as White or another race. These results suggest bolstering resilience may be an important strategy for future interventions aiming to improve ART adherence over time for racially and ethnically diverse SMM with HIV.

## 1. Introduction

### 1.1. HIV Among Sexual Minority Men (SMM) in the United States

SMM are disproportionately impacted by the HIV epidemic, representing 67% of new HIV infections in the United States for 2022 [1]. Further, Black/African American (34.1 infections per 100,000), multiracial (21.6 infections per 100,000), and Hispanic/Latine (20.7 infections per 100,000) groups face higher HIV incidence rates than other racial/ethnic groups [1]. Despite the implementation of public health programs aimed at improving the availability of antiretroviral treatment (ART), critical gaps and racial disparities in ART access, adherence, and viral suppression for SMM persist [2].

### 1.2. Psychosocial Factors and HIV

SMM and people with HIV (PWH) in the United States experience unique psychosocial barriers to engaging in the HIV care continuum. For example, SMM and PWH are diagnosed with depression at 2–3 times the rate of the general population [3, 4], which has been associated with suboptimal ART adherence [3]. Additionally, HIV stigma may exacerbate an already elevated risk for depression and disruptions in HIV care among these groups [5–7]. Further, SMM with HIV report higher rates of substance use [8], and stimulant use, specifically, has been consistently found to impact viral load for PLWH [9, 10]. Increasing trends in U.S. methamphetamine use have been observed (2011–2017) for SMM, with notable increases among Black and Hispanic/Latine SMM during that time [11].

### 1.3. Resilience and HIV

The strengths-based concept of resilience, defined as a dynamic process wherein individuals psychologically, behaviorally, and/or socially adapt to adversity [12], has been identified as potentially important for HIV-related research [12]. For example, research has found negative associations between resilience and depressive symptoms, sexual minority stigma, and substance use among SMM and PWH [13, 14]. Limited research exists, however, exploring the association between resilience and HIV treatment outcomes among SMM. In one exploratory study with SMM in Louisiana, resilience was associated with positive HIV outcomes (i.e., reduced time between HIV care visits), particularly for Black/African American SMM [15]. Additionally, in a cross-sectional study with PWH in South Carolina, depression mediated the relationship between resilience and ART adherence, but only among Black/African American PWH [16]. Collectively, this indicates that resilience could be a critical resource to improve HIV and psychosocial outcomes for SMM [17], particularly for minoritized racial groups [15, 16]. Little is known, however, about the longitudinal relationship among resilience, psychosocial factors (e.g., depression, stigma, and substance use), and HIV-related outcomes for racially diverse SMM.

In contrast to the cross-sectional analyses conducted in prior studies, longitudinal analyses enable us to examine associations over time to determine causality. Moreover, longitudinal analyses can decompose the association between a time-varying predictor and an outcome into separate between- and within-person effects. Using resilience and ART adherence as an example, the between-person effect would estimate the association between participants' average resilience and average ART adherence over time, whereas the within-person effect would estimate the association between a participant's fluctuations in resilience and ART adherence around their own averages. This approach may provide a nuanced understanding of the relationship between resilience and ART adherence, like whether there are critical timepoints for intervention (e.g., when a person's resilience level dips below their average) or whether more global resilience interventions may be effective for improving longitudinal ART outcomes. As such, robust longitudinal approaches are needed to further explore how resilience and other psychosocial factors might impact ART adherence over time for SMM.

### 1.4. The Current Study

This study tested (1) the associations, including between- and within-person effects, between resilience, psychosocial factors, and ART adherence across a 17-month timeframe in a sample of racially diverse SMM with HIV in New York and (2) the moderating effect of race on the longitudinal relationship between resilience and ART adherence.

## 2. Methods

### 2.1. Participants and Procedures

Data were drawn from *Thrive With Me* (TWM), a prospective, randomized controlled trial of an mHealth intervention to improve ART adherence and viral suppression among SMM with HIV through ART information, ART self-monitoring, and peer support [18]. Details about the TWM intervention have previously been published [18–20]. Briefly, TWM recruited 401 SMM with HIV from the New York, NY area beginning in October 2016. Participants were eligible if they reported having a detectable viral load in the past year and/or suboptimal past 30-day ART adherence. Eligible participants were randomized to either a 5-month TWM intervention or an informational control arm. Computer-assisted self-interviewing assessments were conducted in person at the Pride Health Research Consortium (PRIDE) offices at baseline, 5-month, 11-month, and 17-month time points. All participants provided informed consent prior to enrollment, received U.S. $50 for each assessment, and follow-up assessments were completed as of August 2019. Study procedures were approved by the respective IRBs of the University of Minnesota and the Hunter College City University of New York.

### 2.2. Measures

#### 2.2.1. Sociodemographic Characteristics

At the TWM baseline, participants self-reported their sociodemographic factors, including their age (in years), ethnicity (Hispanic/Latine vs. not Hispanic/Latine), race (White, Black/African American, or Other), highest level of education (less than a high school diploma, high school diploma/GED, some college, and college degree), and annual income ($0–19,999, $20,000–39,999, $40,000–74,999, and $75,000+).

#### 2.2.2. Resilience

Resilience was measured across all study timepoints using an adapted, 10-item version of the validated Resilience Scale [21], which asked participants to rate the degree to which they agree/disagree with statements like the following: “My belief in myself gets me through hard times.” All items were summed (range: 10–50), with higher scores indicating higher resilience. The Resilience Scale has consistently demonstrated excellent internal consistency and validity in past research [21]. In the current sample, the shortened 10-item version of the scale demonstrated excellent internal consistency (*α* = 0.91; Ω = 0.93), acceptable convergent validity (moderate-to-high negative correlation with depressive symptoms), and desirable discriminant validity (low correlations with HIV stigma; Supporting Table 1).

#### 2.2.3. Psychosocial Characteristics

Past 7-day depressive symptoms were measured across all study timepoints using the 10-item Center for Epidemiologic Studies Depression Scale (CESD-10) [22]. Items were summed to create a total depressive symptom score (0–30) and then dichotomized, with a score of ≥ 10 indicating depressive symptomology [23]. The CESD-10 demonstrated good reliability in our sample (*α* = 0.80) and in past research [24].

HIV stigma was measured across all study timepoints with the 24-item HIV Stigma Mechanism Scale [5]. The 9-item anticipated HIV stigma subscale assessed participants' expectations of mistreatment related to their HIV status, the 6-item internalized HIV stigma subscale measured participants' self-devaluation related to their HIV status, and the 9-item enacted HIV stigma subscale asked for participants' direct experiences of HIV-related discrimination. Items were reverse coded when appropriate and averaged to create subscale scores. Each subscale demonstrated good reliability in the current sample (*α* = 0.89–0.92) and in previous research [5].

Substance use was evaluated across all study timepoints through the Integrated E-Z Split Key Cup II-5 urine screen panel (Innovation Laboratories) [25], which measured the use of marijuana, cocaine, amphetamines, methamphetamine, and opioids. This panel was able to detect the use of these drugs from 1 to 4 days after use [25]. For the current study, we focused on recent stimulant use (i.e., cocaine, amphetamine, and methamphetamine use), and urine screen results were combined to measure (0) no stimulant use detected versus (1) any cocaine, amphetamine, and/or methamphetamine use detected.

#### 2.2.4. ART Adherence

The outcome of interest, past 30-day ART adherence, was assessed at all study timepoints via the following question: “In the last 30 days, on how many days did you miss at least one dose of any of your ART medications?” Participants' self-reported number of missed ART doses was then dichotomized to represent optimal (≥ 90% of days with no missed doses) versus suboptimal (< 90% of days with no missed doses) ART adherence.

### 2.3. Analyses

Descriptive statistics and correlations were calculated for participant sociodemographic characteristics, resilience, psychosocial factors, and ART adherence at both the between- and within-person levels. Next, generalized estimating equations (GEEs) [26] assessed the longitudinal relationship between resilience, psychosocial factors, and ART adherence. The logit-link function was used for all GEE analyses, and therefore estimated coefficients represent expected changes in log–odds of ART adherence for each 1-unit increase in the predictor (analogous to results from logistic regression). Further, we employed a multivariable model-building approach in which resilience, depressive symptoms, and stimulant use were each initially entered into separate models predicting ART adherence (Models 1–3), then entered simultaneously together (Model 4), and then entered in a model that controlled for sociodemographic characteristics, TWM intervention assignment, and HIV stigma (Model 5). Additional models assessed the moderating effect of race on the relationship between resilience and ART adherence (Model 6), while controlling for the moderating effect of race on the relationship between income and ART adherence (Model 7) and all covariates (Model 8). We chose to include the TWM intervention assignment as a covariate rather than a potential independent variable or moderator of interest in the analyses given that past analyses have indicated that there is no significant relationship between the TWM intervention condition and ART adherence [20].

In order to estimate separate between- and within-person effects, and to be best aligned with standard practice for longitudinal data analysis [27], we separated each time-varying predictor into two variables within each model. Specifically, if *X*_*it*_ and *Y*_*it*_ are a time-varying predictor and outcome for an individual *i* at time point *t* (e.g., raw repeated measures of resilience and ART adherence), then *X*_*it*_ can be separated into ${\overset{¯}{X}}_{i}$ and *X*_*it*_^∗^ (i.e., average resilience across time [between-person effects] and a deviation above/below that average [within-person effects]) such that $X_{it}={\overset{¯}{X}}_{i}+X_{it}^{*}$. Then, both ${\overset{¯}{X}}_{i}$ and *X*_*it*_^∗^ may be used to simultaneously predict *Y*_*it*_ [28]. The same decomposition holds for binary predictors (e.g., stimulant use) [29]. All statistical analyses were conducted using R Version 4.4.1 [30].

## 3. Results

### 3.1. Descriptive Statistics

A total of 401 SMM with HIV completed the TWM baseline interview. Participants were, on average, 39.1 years old (SD = 10.8). Most participants self-identified as Black/African American (59.9%), with fewer identifying as White (29.4%) or another race (10.7%). Mean resilience scores at baseline were 41.2 (SD = 6.8), and 61% of participants reported optimal past 30-day ART adherence (Table 1). Tables 2 and 3 present between- and within-level correlations amongst ART adherence, the predictors of interest, and all covariates.

### 3.2. Unique Association Between Resilience and ART Adherence

Models 1–3, respectively, used resilience, depressive symptoms, and stimulant use to predict ART adherence (at both between- and within-person levels), providing an estimate of the “raw” or “total” association between each predictor and ART adherence (Table 4). Results show that resilience and depressive symptoms predicted ART adherence at both between- and within-person levels, whereas stimulant use only predicted ART adherence at the between-person level.

Model 4 included resilience, depressive symptoms, and stimulant use in a single model to predict ART adherence. Notably, resilience and stimulant use maintained significant prediction at the between-person level, whereas depressive symptoms maintained significant prediction at the within-person level. Model 5 extended Model 4 to include multiple covariates (race, age, TWM intervention assignment, education level, income, and HIV stigma). Importantly, the between- and within-effects of resilience maintained significant prediction of ART adherence, above and beyond the extra covariates added to Model 5.

### 3.3. Race as a Moderator of the Relationship Between Resilience and ART Adherence

In addition to Models 1–5, we examined race as a moderator of the longitudinal association between resilience and ART adherence. Noting that race may moderate any of the remaining predictors in Model 5, and that including all possible interactions in one model is both too complex to interpret and would notably reduce statistical power, we first explored which interactions independently produced a significant prediction of ART adherence (Supporting Table 2). Only two interactions produced significant predictions of ART adherence at the between-person level: Race × Resilience and Race × Income. Therefore, we estimated three more models (Models 6–8; Table 5) to understand the interaction between race and resilience (Model 6), while controlling for the interaction between race and income (Model 7), as well as all other covariates (Model 8).

**Figure 1:**
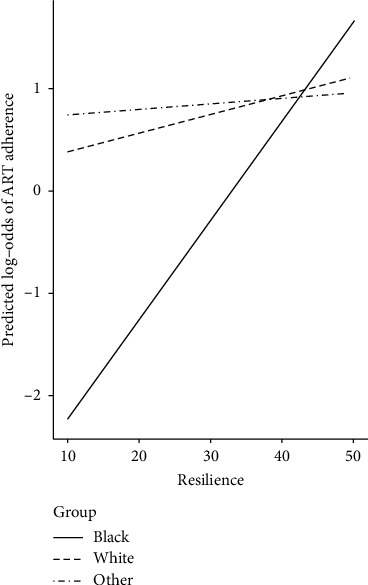
The moderating effect of race on the relationship between resilience and antiretroviral treatment (ART) adherence among a sample of sexual minority men with HIV (*n* = 401).

Model 8 demonstrated that, at the between-person level, and after controlling for the interaction between race and income and all remaining covariates, Black SMM had a more positive longitudinal association between resilience and ART adherence than SMM that identified as White or another race (*b*_Black_ = 0.10, *p* < 0.001; *b*_White_ = 0.02, *p*=0.55; *b*_Other_ = 0.01, *p*=0.90; *b*_Black_ − *b*_White_ = 0.08, *p*=0.02; *b*_Black_ − *b*_Other_ = 0.08, *p*=0.05; Figure 1). No notable differences were observed between those identifying as White and another race (*b*_White_ − *b*_Other_ = 0.01, *p*=0.81). Additionally, at the between-person level, recent stimulant use was negatively associated with ART adherence while controlling for the interaction between race and resilience and all other covariates.

## 4. Discussion

The current study assessed the prospective associations between resilience, psychosocial factors, and ART adherence among a racially diverse sample of SMM with HIV. We found that resilience longitudinally predicted better ART adherence across the 17-month study timeframe. These findings align with, and extend, past cross-sectional research finding resilience is associated with positive HIV care outcomes among SMM [15]. Our findings further expand the scientific literature by providing novel and nuanced insights on the longitudinal relationship between resilience and ART adherence.

Specifically, we found that individuals' average resilience across time (between-person resilience) was a significant predictor of ART adherence among racially diverse SMM. These findings provide critical information to inform HIV-related intervention development among this population. Notably, this indicates that more global resilience interventions may be most effective for improving longitudinal ART-related outcomes among SMM, rather than interventions targeting specific timepoints (e.g., when a person's resilience level dips). While consistent with previous research highlighting depression and stimulant use as critical challenges to ART adherence among SMM [8, 31], this study expands the literature by identifying that resilience predicted ART adherence beyond the effects of depressive symptoms and stimulant use, underscoring the potential benefits of resilience on HIV-related outcomes for this population. As such, these findings demonstrate that resilience-strengthening strategies (e.g., strategies for making services more accessible, attending to socio-cultural and community norms, and providing social support) [32] could be important resources to incorporate into existing HIV treatment services to support longitudinal HIV outcomes for SMM.

Importantly, the current study found that the longitudinal association between resilience and ART adherence was moderated by participants' race. Similar to limited research [15, 16], we found a positive longitudinal association between resilience and ART adherence at the between-person level for those that identified as Black/African American, but not for those that identified as White or another race. These findings provide robust evidence that the longitudinal relationship between resilience and ART adherence may be moderated by race given that this association was retained even when controlling other well-documented factors that impact ART adherence (e.g., depressive symptoms, stimulant use, and HIV stigma) [4–6, 8, 33, 34] and the interaction between race and income. Given the robust nature of these findings, and that these findings mirror results from other exploratory research with racially and ethnically diverse PWH [15, 16], future research should further examine the potential causal pathways between resilience and HIV-related outcomes (e.g., the potential for resilience to lower depression and improve positive coping and self-efficacy) [35] across racial groups to better understand these processes and to identify key intervention targets.

Collectively, these findings indicate that interventions aimed at improving HIV-related outcomes for SMM could potentially benefit from integrating resilience-based materials and supports. As such, it is recommended that interventions targeting resilience among SMM with HIV (e.g., the Brothers Building Brothers by Breaking Barriers intervention) [17, 36] be adopted into existing HIV care and treatment supports, particularly for Black/African American SMM. Additionally, future research should investigate whether these interventions are able to improve ART adherence for racially diverse SMM over extended periods of time, or whether HIV care services will need to periodically provide additional supports to further bolster resilience over time for this group.

### 4.1. Limitations

This study has several limitations to consider. First, though we employed multiple recruitment approaches, the final sample only included SMM with HIV that could attend in-person assessments at the PRIDE offices in New York, NY. Further, the sample was predominately Black/African American (60%), and highly educated (76% had at least some college education). As such, the representativeness of the sample and the generalizability of the findings may be limited (especially given the relatively small sample of those identifying as a race other than White or Black). However, the recruited sample closely resembles the racial/ethnic composition of HIV diagnoses in the United States [2, 37]. In addition, due to the use of a longitudinal design, we acknowledge attrition and the potential biases introduced by resulting missingness are limitations for these analyses. However, a total of 351 (88%), 324 (81%), and 318 (79.3%) of the original 401 participants completed the 5-month, 11-month, and 17-month follow-up surveys, respectively. Though retention rates were within acceptable limits, it is still possible that bias was introduced due to attrition. Further, though we drew from a well-validated measure of resilience [21], we implemented a shortened 10-item version of this scale, thereby potentially limiting the conclusions that can be drawn regarding resilience in this sample. It is important to note, however, that this shortened version of the scale still demonstrated good reliability and validity. Lastly, it is possible that, within our statistical approach for assessing significant interaction effects, we could have both increased the familywise Type 1 error rate (through conducting multiple tests) and missed complex interactions that would emerge when controlling for covariates (thereby increasing our Type II error rate). We believe, however, that our approach offered a good balance to potential Type I and Type II error risk.

## 5. Conclusion

The current study identified that resilience was longitudinally associated with optimal ART adherence across a 17-month timeframe and may be particularly beneficial for SMM with HIV. Specifically, we identified individuals' average resilience across time predicted ART adherence above and beyond the individual effects of depressive symptoms and stimulant use for SMM identifying as Black/African American. The results of this study can lay the foundation for additional research to identify the causal pathways and intervention targets within the relationship between resilience and ART adherence. Further, these findings have important implications for the development of tailored intervention and treatment efforts aimed at enhancing resilience, psychological well-being, and disease progression among racially diverse SMM with HIV.

## Figures and Tables

**Table 1:** Sociodemographic characteristics at baseline among SMM with HIV (*n* = 401).

Variable	*n* (%)
Age (mean, SD)	39.08 (10.8)
Race	
White	113 (29.4)
Black/African American	230 (59.9)
Other	41 (10.7)
Ethnicity	
Hispanic/Latine	108 (27.1)
Not Hispanic/Latine	290 (72.9)
TWM condition	
Intervention	202 (50.4)
Control	199 (49.6)
Resilience score (mean, SD)	41.22 (6.8)
Information score (mean, SD)	36.70 (5.8)
Motivation score (mean, SD)	34.03 (8.4)
Behavioral skills score (mean, SD)	48.20 (8.5)
Depressive symptoms	
Yes	194 (49.1)
No	201 (50.9)
Any recent drug use	
Yes	219 (55.7)
No	174 (44.3)
Recent stimulant use	
Yes	111 (28.1)
No	284 (71.9)
HIV stigma	
Anticipated stigma (mean, SD)	2.01 (0.9)
Internalized stigma (mean, SD)	2.19 (1.1)
Enacted stigma (mean, SD)	1.51 (0.7)
Optimal 30-day ART adherence	
Yes	265 (66.1)
No	136 (33.9)
Detectable viral load	
Yes	154 (38.5)
No	246 (61.5)

Abbreviations: ART, antiretroviral treatment; SD, standard deviation; SMM, sexual minority men.

**Table 2:** Descriptive statistics for between-level ART adherence, resilience, and all covariates within a sample of SMM with HIV (*n* = 401).

	1.	2.	3.	4.	5.	6.	7.	8.	9.	10.	11.	12.	13.
1. ART adherence	1.00												
2. Resilience	0.27^∗∗∗^	1.00											
3. Depressive symptoms	−0.20^∗∗∗^	−0.56^∗∗∗^	1.00										
4. Stimulant use	−0.21^∗∗∗^	−0.12^∗∗∗^	0.09^∗∗∗^	1.00									
5. Race (Black)	−0.03	0.20^∗∗∗^	−0.12^∗∗∗^	0.03	1.00								
6. Race (Other)	0.01	0.01	−0.02	−0.09^∗∗∗^	−0.42^∗∗∗^	1.00							
7. TWM condition	−0.08^∗∗^	−0.03	0.06^∗^	0.09^∗∗∗^	0.08^∗∗^	−0.08^∗∗^	1.00						
8. Age	0.05°	0.07^∗∗^	−0.14^∗∗∗^	0.07^∗∗^	−0.08^∗∗^	−0.11^∗∗∗^	0.09^∗∗∗^	1.00					
9. Education level	0.12^∗∗∗^	0.09^∗∗∗^	−0.07^∗∗^	−0.15^∗∗∗^	−0.32^∗∗∗^	0.11^∗∗∗^	−0.15^∗∗∗^	0.15^∗∗∗^	1.00				
10. Annual income	0.08^∗∗^	0.05^∗^	−0.13^∗∗∗^	−0.13^∗∗∗^	−0.19^∗∗∗^	−0.01	−0.07^∗∗^	0.08^∗∗^	0.35^∗∗∗^	1.00			
11. Anticipated HIV stigma	−0.12^∗∗∗^	−0.35^∗∗∗^	0.40^∗∗∗^	0.08^∗∗^	−0.11^∗∗∗^	0.12^∗∗∗^	−0.01	−0.09^∗∗∗^	0.00	−0.06^∗^	1.00		
12. Internalized HIV stigma	−0.13^∗∗∗^	−0.46^∗∗∗^	0.45^∗∗∗^	0.01	−0.12^∗∗∗^	0.02	−0.01	−0.14^∗∗∗^	−0.04	−0.01	0.57^∗∗∗^	1.00	
13. Enacted HIV stigma	−0.06^∗∗^	−0.21^∗∗∗^	0.33^∗∗∗^	0.15^∗∗∗^	−0.09^∗∗∗^	0.13^∗∗∗^	−0.08^∗∗∗^	−0.04	−0.02	−0.06^∗^	0.73^∗∗∗^	0.36^∗∗∗^	1.00
Mean	0.70	41.27	0.47	0.30	0.60	0.11	0.50	39.08	3.03	1.67	1.97	2.15	1.51
SD	0.36	6.36	0.40	0.38	0.49	0.31	0.50	10.75	0.89	0.93	0.76	1.00	0.62

*Note*: °, ^∗^, ^∗∗^, and ^∗∗∗^, respectively, indicate *p* < 0.10, *p* < 0.05, *p* < 0.01, and *p* < 0.001.

Abbreviations: ART, antiretroviral treatment; SD, standard deviation; SMM, sexual minority men; TWM, *Thrive With Me* intervention.

**Table 3:** Descriptive statistics for within-level ART adherence, resilience, and all covariates among a sample of SMM with HIV (*n* = 401).

	1.	2.	3.	4.	5.	6.	7.	8.
1. ART adherence	1.00							
2. Resilience	0.08^∗∗^	1.00						
3. Depressive symptoms	−0.10^∗∗∗^	−0.24^∗∗∗^	1.00					
4. Stimulant use	−0.02	0.00	0.03	1.00				
5. Study visit number	0.05°	0.00	−0.05°	0.05°	1.00			
6. Anticipated HIV stigma	−0.02	−0.06^∗^	0.09^∗∗∗^	−0.02	−0.05°	1.00		
7. Internalized HIV stigma	−0.07^∗∗^	−0.14^∗∗∗^	0.15^∗∗∗^	−0.05^∗^	−0.06^∗^	0.25^∗∗∗^	1.00	
8. Enacted HIV stigma	0.01	−0.01	0.07^∗∗^	−0.05°	0.00	0.46^∗∗∗^	0.18^∗∗∗^	1.00
Mean	0.00	0.00	0.00	0.00	0.00	0.00	0.00	0.00
SD	0.30	3.81	0.31	0.26	1.12	0.43	0.49	0.35

*Note*: °, ^∗^, ^∗∗^, and ^∗∗∗^, respectively, indicate *p* < 0.10, *p* < 0.05, *p* < 0.01, and *p* < 0.001.

Abbreviations: ART, antiretroviral treatment; SMM, sexual minority men; SD, standard deviation.

**Table 4:** Bivariate and multivariable generalized estimating equation parameter estimates testing the relationship between resilience and ART adherence among a sample of SMM with HIV (*n* = 401).

	Model 1	Model 2	Model 3	Model 4	Model 5
Between					
Resilience	0.07^∗∗∗^ (0.45)			0.05^∗∗∗^ (0.34)	0.06^∗∗∗^ (0.38)
Depressive symptoms (ref = no symptoms)		−0.92^∗∗∗^ (−0.37)		−0.44° (−0.18)	−0.29 (−0.11)
Stimulant use (ref = no use)			−0.82^∗∗∗^ (−0.32)	−0.76^∗∗∗^ (−0.30)	−0.84^∗∗∗^ (−0.32)
Black (ref = White)					−0.22 (−0.11)
Other race (ref = White)					−0.34 (−0.10)
TWM condition (ref = control condition)					−0.20 (−0.10)
Age					0.01 (0.14)
Education level					0.01 (0.01)
Annual income					0.05 (0.05)
Anticipated HIV stigma					−0.17 (−0.13)
Internalized HIV stigma					0.08 (0.08)
Enacted HIV stigma					0.14 (0.09)
Within					
Resilience	0.03^∗^ (0.10)			0.02° (0.09)	0.03^∗^ (0.12)
Depressive symptoms (ref = no symptoms)		−0.48^∗∗∗^ (−0.15)		−0.38^∗^ (−0.12)	−0.33^∗^ (−0.10)
Stimulant use (ref = no use)			−0.18 (−0.04)	−0.16 (−0.04)	−0.07 (−0.02)
Time					0.06 (0.08)
Anticipated HIV stigma					0.01 (< 0.01)
Internalized HIV stigma					−0.16 (−0.08)
Enacted HIV stigma					0.13 (0.04)

*Note*: °, ^∗^, ^∗∗^, and ^∗∗∗^, respectively, indicate *p* < 0.10, *p* < 0.05, *p* < 0.01, and *p* < 0.001. Values in parentheses indicate standardized estimates.

Abbreviations: ART, antiretroviral treatment; SMM, sexual minority men; TWM, *Thrive With Me* intervention.

**Table 5:** Bivariate and multivariable generalized estimating equation parameter estimates testing the relationship between resilience and ART adherence among a SMM with HIV (*n* = 401).

	Model 6	Model 7	Model 8
Between			
Resilience (White)	0.04° (0.25)	0.04 (0.23)	0.02 (0.12)
Resilience × Black	0.07^∗^ (0.32)	0.07^∗^ (0.34)	0.08^∗^ (0.37)
Resilience × Other race	−0.02 (−0.04)	−0.03 (−0.06)	−0.01 (−0.03)
Depressive symptoms (ref = no symptoms)			−0.27 (−0.11)
Stimulant use (ref = no use)			−0.82^∗∗∗^ (−0.31)
Black (ref = White)	−0.26 (−0.13)	−0.16 (−0.08)	−0.14 (−0.07)
Other race (ref = White)	−0.17 (−0.05)	−0.01 (−0.00)	−0.04 (−0.01)
TWM condition (ref = control condition)			−0.15 (−0.08)
Age			0.01 (0.09)
Education level			0.87 (0.02)
Income (White)		0.32^∗^ (0.30)	0.26 (0.25)
Income × Black		−0.58^∗∗^ (−0.34)	−0.58^∗∗^ (−0.34)
Income × Other race		0.45 (0.13)	0.50 (0.15)
Anticipated HIV stigma			−0.18 (−0.13)
Internalized HIV stigma			0.12 (0.12)
Enacted HIV stigma			0.07 (0.04)
Within			
Resilience			0.03^∗^ (0.12)
Depressive symptoms (ref = no symptoms)			−0.34^∗^ (−0.11)
Stimulant use (ref = no use)			−0.08 (−0.02)
Time			0.07 (0.07)
Anticipated HIV stigma			0.01 (< 0.01)
Internalized HIV stigma			−0.17 (−0.08)
Enacted HIV stigma			0.12 (0.04)

*Note*: °, ^∗^, ^∗∗^, and ^∗∗∗^, respectively, indicate *p* < 0.10, *p* < 0.05, *p* < 0.01, and *p* < 0.001. Values in parentheses indicate standardized estimates.

Abbreviations: ART, antiretroviral treatment; SMM, sexual minority men; TWM, *Thrive With Me* intervention.

## Data Availability

The data that support the findings of this study are available from the corresponding author upon reasonable request.

## Supporting Information

Supporting Information 1 Supporting Table 1: This table provides the results of analyses assessing the internal consistency and the convergent and discriminant validity of the shortened Resilience Scale in a sample of racially and ethnically diverse sexual minority men with HIV (*n* = 401).

Supporting Information 2 Supporting Table 2: This table provides the results of analyses evaluating whether any interactions between race and the predictors of interest (i.e., resilience, depressive symptoms, stimulant use, intervention, age, education level, annual income, treatment condition, anticipated HIV stigma, internalized HIV stigma, and enacted HIV stigma) produced a significant prediction of ART adherence. All interactions were assessed at both the between- and within-person levels.
