# Supplementary material for: Resilience and ART Adherence Among a Sample of Racially and Ethnically Diverse Sexual Minority Men With HIV
Source: AIDS Res Treat. 2025 Aug 12;2025:8199608. doi: 10.1155/arat/8199608 (PMC12364592; doi:10.1155/arat/8199608)
Supplement: Supporting Information 1 — Supporting Table 1: This table provides the results of analyses assessing the internal consistency and the convergent and discriminant validity of the shortened Resilience Scale in a sample of racially and ethnically diverse sexual minority men with HIV (n = 401). [file 8199608.f1.docx]

**Supplemental Table 1.** The Reliability and Convergent and Discriminant Validity of the Resilience Scale in a sample of SMM with HIV (*n* = 401).

| ***Reliability of the Resilience Scale*** | | | | | |
| --- | --- | --- | --- | --- | --- |
|  | **Revelle’s Omega (Ω)** | | | | **Cronbach’s Alpha (α)** |
| *Baseline* | 0.93 | | | | 0.91 |
| *5-Month Follow-Up* | 0.95 | | | | 0.93 |
| *11-Month Follow-Up* | 0.99 | | | | 0.98 |
| *17-Month Follow-Up* | 0.99 | | | | 0.99 |
| ***Convergent and Discriminant Validity of the Resilience Scale*** | | | | | |
| **Measure** | **1** | **2** | **3** | **4** | **5** |
| **1.** Resilience Scale | 1 | –0.58 | –0.44 | –0.25 | –0.17 |
| **2.** Depression Symptoms |  | 1 | 0.46 | 0.35 | 0.27 |
| **3.** Internalized HIV Stigma |  |  | 1 | 0.52 | 0.29 |
| **4.** Anticipated HIV Stigma |  |  |  | 1 | 0.65^†^ |
| **5.** Enacted HIV Stigma |  |  |  |  | 1 |

*SMM: Sexual Minority Men. Note: Numbers highlighted in green represent convergent validity. Numbers highlighted in blue represent discriminant validity.* ^†^Subscales are highly correlated and may produce issues of multicollinearity.
