# Supplementary material for: Resilience and ART Adherence Among a Sample of Racially and Ethnically Diverse Sexual Minority Men With HIV
Source: AIDS Res Treat. 2025 Aug 12;2025:8199608. doi: 10.1155/arat/8199608 (PMC12364592; doi:10.1155/arat/8199608)
Supplement: Supporting Information 2 — Supporting Table 2: This table provides the results of analyses evaluating whether any interactions between race and the predictors of interest (i.e., resilience, depressive symptoms, stimulant use, intervention, age, education level, annual income, treatment condition, anticipated HIV stigma, internalized HIV stigma, and enacted HIV stigma) produced a significant prediction of ART adherence. All interactions were assessed at both the between- and within-person levels. [file 8199608.f2.docx]

**Supplemental Table 2.** Examining Differences in the Associations between Resilience, ART Adherence, and Covariates across Racial Groups among SMM with HIV (*n* = 401).

|  |  | **White** | **Black** | **Other Race** | **Black vs White** | **Other vs. White** | **Black vs Other Race** | **Joint Test** |
| --- | --- | --- | --- | --- | --- | --- | --- | --- |
| **Between** |  |  |  |  |  |  |  |  |
|  | Resilience | .04^°^ (.14) | .11^***^ (.50) | .02 ( .04) | .07^*^ (.36) | -.02 (-.09) | .09^*^ (.45) | * |
|  | Depressive Symptoms | -.53 (-.12) | -1.1^***^ (-.35) | -.50 (-.07) | -.59 (-.23) | .04 (.05) | -.63 (-.28) |  |
|  | Stimulant Use | -.92^*^(-.19) | -1.0^***^ (-.30) | -.41 (-.04) | -.08 (-.11) | .50 (.15) | -.59 (-.26) |  |
|  | TWM Treatment Condition | -.22 (-.06) | -.33 (-.13) | .27 ( .04) | -.11 (-.07) | .49 (.10) | -.59 (-.17) |  |
|  | Age | .01 (.05) | .01 (.09) | .05 ( .20) | .00 (.03) | .04 (.15) | -.04 (-.11) |  |
|  | Education Level | .06 (.03) | .18 (.13) | .63^°^ ( .18) | .12 (.10) | .57 (.15) | -.45 (-.05) |  |
|  | Annual Income | .33^°^ (.21) | -.14 (-.08) | .78^**^ ( .23) | -.46^°^ (-.29) | .46 (.02) | -.92^**^ (-.31) | ** |
|  | Anticipated HIV Stigma | -.17 (-.07) | -.39^**^ (-.24) | .03 ( .01) | -.22 (-.17) | .21 (.08) | -.43 (-.24) |  |
|  | Internalized HIV Stigma | .05 (.03) | -.37^***^ (-.27) | -.21 (-.07) | -.42^*^ (-.30) | -.26 (-.10) | -.16 (-.21) | ° |
|  | Enacted HIV Stigma | -.13 (-.04) | -.23 (-.11) | -.06 (-.01) | -.10 (-.07) | .08 (.03) | -.18 (-.10) |  |
| **Within** |  |  |  |  |  |  |  |  |
|  | Resilience | .04^**^ (.09) | .03^*^ (.09) | .03 (.04) | -.01 (.00) | -.01 (-.05) | .00 (.04) |  |
|  | Depressive Symptoms | -.40^°^ (-.07) | -.59^***^ (-.14) | -.33 (-.03) | -.19 (-.08) | .07 (.03) | -.27 (-.11) |  |
|  | Stimulant Use | -.02 (.00) | .00 (.00) | -.86 (-.08) | .02 (.00) | -.84 (-.08) | .86 (.08) |  |
|  | Time | .02 (.01) | .12^*^ (.12) | .07 (.03) | .11 (.11) | .05 (.01) | .05 (.09) |  |
|  | Anticipated HIV Stigma | -.12 (-.03) | -.05 (-.02) | -.21 (-.03) | .07 (.01) | -.09 (.00) | .16 (.01) |  |
|  | Internalized HIV Stigma | -.24 (-.06) | -.30^*^ (-.12) | .32 (.05) | -.07 (-.05) | .56^°^ (.12) | -.62^*^ (-.17) | ° |
|  | Enacted HIV Stigma | -.40^°^ (-.08) | .20 (.05) | .56 (.07) | .60^*^ (.14) | .96^°^ (.15) | -.36 (-.02) | ° |

*ART: Antiretroviral Treatment; SMM: Sexual Minority Men; TWM: Thrive With Me intervention; Note*: ^°^, ^*^, ^**^, and ^***^ respectively indicate *p* < .10, *p* < .05, *p* < .01, and *p* < .001. Values in parentheses indicate standardized estimates.
